# Influence of different learning modalities on academic and psychological status of nursing university student

**DOI:** 10.1590/1518-8345.0000.4536

**Published:** 2025-07-11

**Authors:** Marta San-Antolín, César Calvo-Lobo

**Affiliations:** 1 Universidad de Valladolid, Facultad de Educación y Trabajo Social, Departamento de Psicología, Valladolid, Spain.; 2 Universidad Complutense de Madrid, Facultad de Enfermería, Fisioterapia y Podología, Madrid, Spain.

**Figure:**
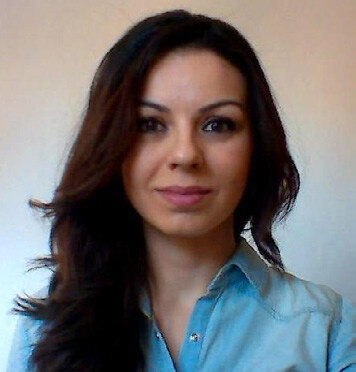

**Figure:**
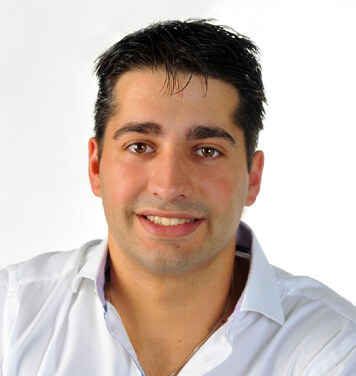

The present editorial aims to detail the influence of different existing education methodologies regarding the academic and psychological status of nursing students during university learning. Different nursing learning methodologies were described in various universities. These methodologies included face-to-face and online learning, problem-based learning, virtual reality, transformative learning, clinical simulation, flipped classroom, and other novel teaching modalities^(1-2)^.

Recent scenarios, such as pandemic environments, have led to increase the use of different learning modalities including virtual learning education as well as hybrid modalities, which combined online classes with face-to-face learning education. Nursing students who received isolated online classes suffered from higher levels of depression that could be secondary to the lack of face-to-face relationships with their classmates or teachers, while students who received hybrid education combining face-to-face and online learning modalities received a direct interaction with their peers or teachers. Consequently, nursing students who received hybrid education associated less depression levels compared to nursing students who received isolated virtual education^(1)^. In line with these findings, another research study determined that the isolated online learning modality worsened the mental health of university students, whereas the hybrid education modality provided a beneficial mental health effect on university students who were more likely to apply positive and active coping strategies, helping to control the negative thoughts as well as minimizing the mental health impairment^(3)^.

Despite active methodologies for university teaching have been widely recommended and used in the teaching-learning process for several years in the fields of Nursing and Health Sciences showing beneficial effects for academic and psychological features of these university students, the use of these active methodologies still requires that nursing teachers and students optimized their benefits improving the implementation of these education modalities and minimizing some factors that have interfered negatively in the traditional higher education environments, such as emotional status, psychological problems, and pedagogical motivation, among others^(2)^. Thus, the following learning active modalities have been widely used in nursing higher education: Problem-Based Learning, Team Learning, Peer Instruction, Tailored Teaching, Think Pair Share, the Four Corners Strategy, the D2R methodology of the Flipped Classroom, Gamification, Active Jigsaw methodology, Microteaching, and Realistic Simulation. Among these active methodologies, the use of clinical simulation in university nursing represents one of the most important hot spots in the active higher education modalities^(2,4-5)^.

Realistic Simulation may be considered as an active methodology that can promote the dynamization of teaching and aims to promote autonomy as well as cognitive and psychomotor skills of nursing students. This educational resource may be capable of promoting a new paradigm compared to traditional teaching in the field of nursing and health sciences. This method allows the nursing student to explore a novel phenomenon or procedure by adjusting the parameters and observing the obtained response, including very different educational experiences described by four levels: medium of instruction, modality of simulation, method of instruction and presentation^(2,4)^. Consequently, the pedagogical design framework of Realistic Simulation presented these four progressive levels that described the educational intervention. Lastly, the simulation modality may be defined as the broad description of the simulation experience and includes four modalities, namely: 1) computer simulation, 2) simulated patient, 3) simulated clinical immersion, and 4) procedural simulation, as well as mixed and hybrid simulations^(4)^.

According to the International Nursing Association for Clinical Simulation and Learning (INACSL), the INACSL Standards of Best Practice Simulation^SM^ consists of standards for best practices in conducting clinical simulation that should be followed in nursing simulation education to ensure an effective assessment and simulated intervention, improving the ability of nursing students to understand the objectives or expected outcomes, and promoting the efficient use of these resources for simulation activities^(2,5)^.

Thus, the current literature presented a wide variety of educational methodologies which showed promising results in different face-to-face and virtual scenarios to improve academic and psychological status during theoretical and clinical learning for nursing university students^(1-2)^.

To conclude, authors emphasize that the use of hybrid and active education methodologies should be standardized and generalized in nursing higher education, combining the use of virtual and face-to-face classes and promoting the teacher´s role as a mediator or facilitator, due to these educational strategies improved mental health as well as increase the motivation, coping and resources of nursing students. Indeed, realistic simulation seems to be a hot spot among active education methodologies with different modalities which should be included in the curricular design of nursing degrees.
