# Supplementary material for: Influence of different learning modalities on academic and psychological status of nursing university student
Source: Rev Lat Am Enfermagem. 2025 Jul 11;33:e4536. doi: 10.1590/1518-8345.0000.4536 (PMC12259215; doi:10.1590/1518-8345.0000.4536)
Supplement: Translation

# Translation: Influência de diferentes modalidades de aprendizagem no estado acadêmico e psicológico de estudantes universitários de enfermagem

**DOI:** 10.1590/1518-8345.0000.4537

**Authors:** Marta San-Antolín, César Calvo-Lobo

**Affiliations:** 1 Universidad de Valladolid, Facultad de Educación y Trabajo Social, Departamento de Psicología, Valladolid, Espanha.; 2 Universidad Complutense de Madrid, Facultad de Enfermería, Fisioterapia y Podología, Madrid, Espanha.

Este editorial tem como objetivo detalhar a influência de diferentes metodologias de educação existentes em relação ao estado acadêmico e psicológico dos estudantes de enfermagem durante a graduação. Diferentes metodologias de aprendizagem de enfermagem foram descritas em várias universidades. Essas metodologias incluem aprendizagem presencial e *online*, aprendizagem baseada em problemas, realidade virtual, aprendizagem transformadora, simulação clínica, sala de aula invertida e outras modalidades de ensino originais^(1-2)^.

Cenários recentes, como pandemias, levaram ao aumento do uso de diferentes modalidades de aprendizagem, incluindo a educação virtual, bem como modalidades híbridas, que combinam aulas *online* com educação presencial. Os alunos de enfermagem que receberam aulas *online* isoladas sofreram níveis mais altos de depressão, que podem vir da falta de relacionamentos presenciais com seus colegas ou professores; enquanto os alunos que receberam educação híbrida, combinando modalidades de aprendizado presencial e *online*, tiveram uma interação direta com seus colegas ou professores. Consequentemente, os estudantes de enfermagem que receberam educação híbrida mostraram níveis de depressão menores em comparação com os estudantes de enfermagem que receberam educação virtual isolada^(1)^. Em paralelo com essas descobertas, outro estudo de pesquisa determinou que a modalidade de aprendizagem *online* isolada piorou a saúde mental dos estudantes universitários, enquanto a modalidade de educação híbrida proporcionou um efeito benéfico para a saúde mental deles, que eram mais propensos a aplicar estratégias de enfrentamento positivas e ativas, ajudando a controlar os pensamentos negativos e a minimizar o comprometimento da saúde mental^(3)^.

Apesar de as metodologias ativas para o ensino universitário serem amplamente recomendadas e utilizadas no processo de ensino-aprendizagem há vários anos nas áreas de Enfermagem e Ciências da Saúde, demonstrando efeitos benéficos para as características acadêmicas e psicológicas desses estudantes universitários, o uso dessas metodologias ativas ainda requer que os professores e estudantes de enfermagem otimizem seus benefícios, melhorando a implementação dessas modalidades de educação e minimizando alguns fatores que têm interferido negativamente nos ambientes tradicionais de ensino superior, como estado emocional, problemas psicológicos e motivação pedagógica, entre outros^(2)^. Assim, as seguintes modalidades ativas de aprendizagem têm sido amplamente utilizadas no ensino superior de enfermagem: Aprendizagem Baseada em Problemas, Aprendizagem em Equipe, Instrução por Pares, Ensino Personalizado, *Think Pair Share*, a Estratégia *Four Corners*, a metodologia D2R da Sala de Aula Invertida, Gamificação, metodologia ativa *Jigsaw*, *Microteaching* e Simulação Realística. Entre essas metodologias ativas, o uso da simulação clínica na enfermagem universitária representa um dos pontos mais importantes nas modalidades ativas de ensino superior^(2,4-5)^.

A Simulação Realística pode ser considerada uma metodologia ativa que estimula a dinamização do ensino e visa promover a autonomia e as habilidades cognitivas e psicomotoras dos alunos de enfermagem. Esse recurso educacional pode ser capaz de promover um novo paradigma em comparação com o ensino tradicional no campo da enfermagem e das ciências da saúde. Esse método permite que o estudante de enfermagem explore um novo fenômeno ou procedimento, ajustando os parâmetros e observando a resposta obtida, incluindo experiências educacionais muito diferentes descritas em quatro níveis: meio de instrução, modalidade de simulação, método de instrução e apresentação^(2,4)^. Consequentemente, a estrutura pedagógica da Simulação Realística apresentou esses quatro níveis progressivos que descrevem a intervenção educacional. Por fim, a modalidade de simulação pode ser definida como a descrição ampla da experiência de simulação e inclui quatro modalidades, a saber: 1) simulação computadorizada, 2) paciente simulado, 3) imersão clínica simulada e 4) simulação de procedimentos, bem como simulações mistas e híbridas^(4)^.

De acordo com a *International Nursing Association for Clinical Simulation and Learning* (INACSL), os INACSL *Standards of Best Practice Simulation* ^SM^ consistem em padrões de melhores práticas na realização de simulação clínica que devem ser seguidos no ensino de simulação de enfermagem para garantir uma avaliação e intervenção simulada eficazes, melhorando a capacidade dos alunos de enfermagem de compreender os objetivos ou resultados esperados e promovendo o uso eficiente desses recursos para atividades de simulação^(2,5)^.

Assim, a literatura atual apresentou uma ampla variedade de metodologias educacionais que mostraram resultados promissores em diferentes cenários presenciais e virtuais para melhorar o estado acadêmico e psicológico durante o aprendizado teórico e clínico de estudantes universitários de enfermagem^(1-2)^.

Em conclusão, os autores enfatizam que o uso de metodologias de educação híbrida e ativa deve ser padronizado e generalizado no ensino superior de enfermagem, combinando o uso de aulas virtuais e presenciais e promovendo o papel do professor como mediador ou facilitador, pois essas estratégias educacionais melhoram a saúde mental e aumentam a motivação, o enfrentamento e os recursos dos estudantes de enfermagem. De fato, a Simulação Realística parece ser um ponto de destaque entre as metodologias de educação ativa com diferentes modalidades que devem ser incluídas no projeto curricular dos cursos de enfermagem.

# Translation: Influencia de diferentes modalidades de aprendizaje en el estado académico y psicológico del estudiante universitario de enfermería

**DOI:** 10.1590/1518-8345.0000.4535

**Authors:** Marta San-Antolín, César Calvo-Lobo

**Affiliations:** 1 Universidad de Valladolid, Facultad de Educación y Trabajo Social, Departamento de Psicología, Valladolid, España.; 2 Universidad Complutense de Madrid, Facultad de Enfermería, Fisioterapia y Podología, Madrid, España.

El presente editorial tiene como objetivo detallar la influencia de diferentes metodologías educativas existentes con respecto al estado académico y psicológico de los estudiantes de enfermería durante el aprendizaje universitario. Se describieron diferentes metodologías de aprendizaje de enfermería en diversas universidades. Estas metodologías incluyeron aprendizaje presencial y en línea, aprendizaje basado en problemas, realidad virtual, aprendizaje transformador, simulación clínica, aula invertida y otras modalidades de enseñanza novedosas^(1-2)^.

Escenarios recientes, como entornos pandémicos, han llevado a aumentar el uso de diferentes modalidades de aprendizaje, incluida la educación virtual, así como las modalidades híbridas,que combinan clases en línea con educación presencial. Los estudiantes de enfermería que recibieron clases en línea de forma aislada presentaron niveles más altos de depresión, lo que podría deberse a la falta de relaciones presenciales con sus compañeros o profesores, mientras que los estudiantes que recibieron una educación híbrida, que combina modalidades de aprendizaje presencial y en línea, tuvieron una interacción directa con sus compañeros o profesores. En consecuencia, los estudiantes de enfermería que recibieron una educación híbrida presentaron niveles de depresión más bajos en comparación con aquellos que recibieron una educación virtual aislada^(1)^. En línea con estos hallazgos, otro estudio de investigación determinó que la modalidad de aprendizaje en línea de forma aislada empeoró la salud mental de los estudiantes universitarios, mientras que la modalidad de educación híbrida tuvo un efecto beneficioso en la salud mental de los estudiantes universitarios, quienes mostraron una mayor tendencia a aplicar estrategias de afrontamiento positivas y activas, lo que ayudó a controlar los pensamientos negativos y a minimizar el deterioro de la salud mental^(3)^.

A pesar de que las metodologías activas para la enseñanza universitaria han sido ampliamente recomendadas y utilizadas desde hace varios años en el proceso de enseñanza-aprendizaje en los campos de la Enfermería y las Ciencias de la Salud, mostrando efectos beneficiosos en aspectos académicos y psicológicos de estos estudiantes universitarios, su uso todavía requiere que tanto los docentes como los estudiantes de enfermería optimicen sus beneficios, mejorando la implementación de estas modalidades educativas y minimizando algunos factores que han interferido negativamente en los entornos tradicionales de educación superior, como el estado emocional, los problemas psicológicos y la motivación pedagógica, entre otros^(2)^. Así, las siguientes modalidades activas de aprendizaje han sido ampliamente utilizadas en la educación superior en enfermería: el Aprendizaje Basado en Problemas, el Aprendizaje en Equipo, la Instrucción entre Pares, la Enseñanza Personalizada, la estrategia *Think-Pair-Share*, la estrategia *Four Corners*, la metodología D2R del Aula Invertida, la Gamificación, la metodología Activa *Jigsaw*, la Microenseñanza y la Simulación Realista. Entre estas metodologías activas, el uso de la simulación clínica en la formación universitaria en enfermería representa uno de los puntos clave más relevantes dentro de las modalidades activas de educación superior^(2,4-5)^.

La simulación realista puede considerarse como una metodología activa que puede promover la dinamización de la docencia y tiene como objetivo promover la autonomía, así como las habilidades cognitivas y psicomotoras de los estudiantes de enfermería. Este recurso educativo puede ser capaz de promover un nuevo paradigma en comparación con la enseñanza tradicional en el campo de la enfermería y las ciencias de la salud. Este método le permite al estudiante de enfermería explorar un fenómeno o procedimiento novedoso ajustando los parámetros y observando la respuesta obtenida, incluyendo experiencias educativas muy diversas que se describen en cuatro niveles: medio de instrucción, modalidad de simulación, método de instrucción y forma de presentación^(2,4)^. En consecuencia, el marco de diseño pedagógico de Simulación Realista presentó estos cuatro niveles progresivos que describieron la intervención educativa. Por último, la modalidad de simulación puede definirse como la descripción amplia de la experiencia de simulación e incluye cuatro modalidades, a saber: 1) simulación por computadora, 2) paciente simulado, 3) inmersión clínica simulada y 4) simulación de procedimientos, así como simulaciones mixtas e híbridas^(4)^.

Según la *International Nursing Association for Clinical Simulation and Learning* (INACSL), los INACSL *Standards of Best Practice: Simulation* ^SM^ consisten en estándares de mejores prácticas para la realización de simulaciones clínicas que deben seguirse en la educación en simulación de enfermería, con el fin de garantizar una evaluación e intervención simulada efectivas, mejorar la capacidad de los estudiantes de enfermería para comprender los objetivos o resultados esperados y promover el uso eficiente de estos recursos en las actividades de simulación^(2,5)^.

Así, la literatura actual presentó una amplia variedad de metodologías educativas que mostraron resultados prometedores en diferentes escenarios presenciales y virtuales para mejorar el estatus académico y psicológico durante el aprendizaje teórico y clínico de estudiantes universitarios de enfermería^(1-2)^.

Para concluir, los autores enfatizan que el uso de metodologías educativas híbridas y activas debería estandarizarse y generalizarse en la educación superior en enfermería, combinando el uso de clases virtuales y presenciales, y promoviendo el papel del docente como mediador o facilitador, ya que estas estrategias educativas mejoran la salud mental, además de aumentar la motivación, el afrontamiento y los recursos de los estudiantes de enfermería. En efecto, la simulación realista parece ser un punto clave entre las metodologías activas de enseñanza, con diferentes modalidades que deberían incluirse en el diseño curricular de los grados en enfermería.
